# Tuning the Microenvironment of Water Confined in Ti_3_C_2_T_*x*_ MXene by Cation Intercalation

**DOI:** 10.1021/acs.jpcc.4c00247

**Published:** 2024-02-14

**Authors:** Mailis Lounasvuori, Teng Zhang, Yury Gogotsi, Tristan Petit

**Affiliations:** † Nanoscale Solid−Liquid Interfaces, Helmholtz-Zentrum Berlin für Materialien und Energie GmbH, Albert-Einstein-Str. 15, 12489 Berlin, Germany; ‡ A.J. Drexel Nanomaterials Institute and Department of Materials Science and Engineering, Drexel University, 3141 Chestnut Street, Philadelphia, Pennsylvania 19104, United States

## Abstract

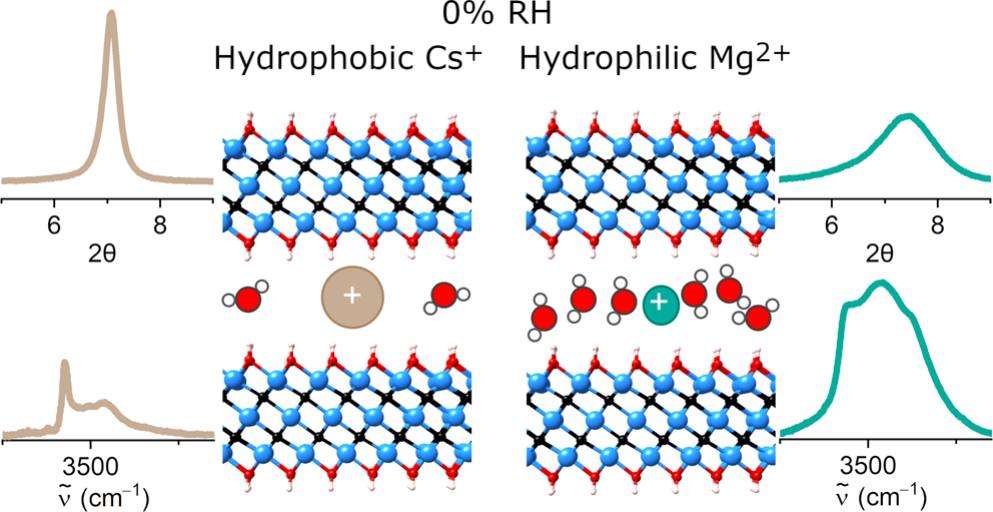

The local microenvironment has recently been found to play a major role in the electrocatalytic activity of nanomaterials. Modulating the microenvironment by adding alkali metal cations into the electrolyte can be used to either suppress hydrogen or oxygen evolution, thereby extending the electrochemical window of energy storage systems, or to tune the selectivity of electrocatalysts. MXenes are a large family of two-dimensional transition metal carbides, nitrides, and carbonitrides that have shown potential for use in electrochemical energy storage applications. Due to their negatively charged surfaces, MXenes can accommodate cations and water molecules between the layers. Nevertheless, the nature of the aqueous microenvironment in the MXene interlayer space is poorly understood. Here, we apply Fourier transform infrared spectroscopy (FTIR) to probe the hydrogen bonding of intercalated water in Ti_3_C_2_T_*x*_ as a function of intercalated cation and relative humidity. Substantial changes in the FTIR spectra after cation exchange demonstrate that the hydrogen bonding of water molecules confined between the MXene layers is strongly cation-dependent. Furthermore, the IR absorbance of the confined water correlates with resistivity estimated by 4-point probe measurements and interlayer distance calculated from XRD patterns. This work demonstrates that cation intercalation strongly modulates the confined microenvironment, which can be used to tune the activity or selectivity of electrochemical reactions in the interlayer space of MXenes in the future.

## Introduction

Modulating the aqueous microenvironment at electrochemical interfaces is vitally important for improving the performance of energy storage materials and electrocatalysts.^1^ The electrochemical window of aqueous batteries^2^ can be extended by adding chemical species to the electrolyte that form strong hydrogen bonds with water molecules,^3−5^ while weakening the H-bonds of water may increase the activity for HER.^6^ Cations are known to tune the selectivity and reaction pathway of electrochemical reactions as shown by the effect of alkali metal cations on product selectivity during electrochemical CO_2_ reduction.^7−10^

Considering the various ways cations may affect electrochemical processes, a better systematic understanding of the role of cations in the aqueous microenvironment is necessary. Ion–water interactions in bulk solutions are generally discussed in terms of the Hofmeister series^11^ that divides cations and anions into structure-making and structure-breaking ions based on their ability to structure water molecules beyond their first solvation shell. While numerous experimental and theoretical studies have been devoted to exploring the solvation structure of ions in the bulk,^12,13^ less is known about confined environments. Theoretical calculations show that ions undergo dehydration and distortion of their hydration shells when confined in a two-dimensional slit,^14,15^ and that the degree of dehydration upon confinement is much greater for weakly hydrated cations such as K^+^ and Cs^+^.^16^

MXenes, a family of 2-dimensional transition metal carbides, nitrides, and carbonitrides first reported in 2011,^17^ have shown potential for electrochemical energy storage and catalysis due to their layered structure, tunable surface chemistry, and high electrical conductivity.^18,19^ They have a general formula of M_*n*+1_X*_n_*T_*x*_, where M is a transition metal, X is carbon or nitrogen, and T_*x*_ represents the heterogeneous surface functionalities (-O, -OH, -F, -Cl, etc.) that are introduced during synthesis. Due to the hydrophilic surface resulting from aqueous etching, MXenes offer an ideal platform to investigate H-bonding in 2D confinement, as shown in our previous work.^20,21^ The interlayer spacing of layered 2D materials, such as MXenes, MoS_2_, and layered double hydroxides, can have an impact on their electrocatalytic activity.^19,22,23^

While the impact of intercalated cations on conductivity^24−26^ and capacitance^27−29^ of MXenes has been investigated, previous studies predominantly used X-ray diffraction (XRD)^24,25,30^ or resistivity measurements^30,31^ and provided limited insights into the water microenvironment. The interlayer spacing revealed by XRD can be used to determine the number of water layers with the help of DFT simulations.^32,33^ However, these methods do not directly show the water’s structural role. The interlayer space varies depending on the surface functionalities, the amount of intercalated water, and the size of the intercalated cation in the case of a fully dried MXene film. XRD studies of multilayered Ti_3_C_2_T_*x*_ with various intercalated cations as a function of humidity have found a discontinuous shift of the *d*-spacing, pointing to mono- and bilayer structures of water.^25,30^ The hydration enthalpy of the cation was determined to be the driving force for this behavior, since the humidity at which the shift occurred varied from cation to cation, and K^+^ was found to not be able to support two water layers inside the structure.^25^

The ions and water intercalated between the MXene layers also affect the electrical resistance of the MXene films. The resistance of Li-intercalated Ti_3_C_2_T_*x*_ was found to increase substantially with humidity, displaying a stepwise pattern that correlates with basal spacing expansion and the relative abundance of two water layers in the interlayer space.^30^ In contrast, when intercalated Li was removed from Ti_3_C_2_T_*x*_ by acid-washing, the electrical resistance exhibited negligible variation of less than 1% in the range of 0–95% RH.^31^ This study did not, however, report on the evolution of the XRD pattern, leaving a gap in the full understanding of structural dynamics under varying humidity levels. Water content in Ti_3_C_2_T_*x*_ films intercalated with various cations was also quantified with thermogravimetric analysis. Ti_3_C_2_T_*x*_ intercalated with a multivalent, high-charge-density cation (Ca^2+^) was found to lose much more water when heated from 27 to 40 °C compared to Li^+^, Na^+^, and K^+^. Monovalent cations exhibited very similar water loss.^25^

All of the above-mentioned reports have only indirectly probed the intercalated water in Ti_3_C_2_T_*x*_ MXene. Spectroscopic methods that are sensitive to water, such as inelastic neutron scattering (INS) or Fourier transform infrared (FTIR) spectroscopy, can elucidate not just relative amounts but also the hydrogen-bonding states of water. Inelastic neutron scattering study of vacuum-annealed, multilayered, intercalated Ti_3_C_2_T_*x*_ found only small amounts of water intercalated with the cations with an increasing degree of ordering in the order Li^+^ < Na^+^ < K^+^.^34^ Temperature-dependent diffuse reflectance FTIR spectroscopy (DRIFTS) has been used to confirm the removal of intercalated water from multilayer Ti_3_C_2_T_*x*_, but the effect of different ions was not investigated and the hydrogen-bonding state of the intercalated water was not discussed.^35^ We have previously demonstrated that FTIR in the attenuated total reflectance (ATR) mode can be used to probe intercalated water in Ti_3_C_2_T_*x*_ with a high signal-to-noise ratio, and we reported on the potential-induced co-intercalation of water with different cations into Ti_3_C_2_T_*x*_ and discovered distinct spectral signatures of water in the hydration shell of Li^+^ and protons.^20,21^

Here, we probe the structure of confined water as a function of intercalated cations and relative humidity using *in situ* FTIR spectroscopy. FTIR spectroscopy is highly sensitive to different H-bonding states of water, especially in the O–H stretching mode region. Because anions do not intercalate into MXene,^36^ this technique allows us to probe the hydration shell around isolated cations in a 2D confined environment. The spectroscopic measurements are complemented with resistivity and interlayer spacing determined under similar conditions. We used LiCl-delaminated Ti_3_C_2_T_*x*_, exchanged Li^+^ for Na^+^, K^+^, Cs^+^, and Mg^2+^, and measured a cation-free film obtained through acid treatment. The identity of the cation was found to greatly influence both the amount and the H-bonding state of water confined within the MXene film.

## Experimental Methods

### Materials

All salt solutions and MXene suspensions were prepared with ultrapure water (Millipore, resistivity 18.2 MΩ·cm). HCl, NaCl, KCl, CsCl, and MgCl_2_ (Carl Roth) were used as received.

### MXene Synthesis

Ti_3_C_2_T_*x*_ MXene was synthesized according to a previously reported procedure.^37^ By including excess aluminum during the synthesis of the Ti_3_AlC_2_ MAX phase precursor, single- and few-layer Ti_3_C_2_T_*x*_ MXene was obtained with improved stoichiometry, resistance to oxidation, and increased electrical conductivity. Briefly, the MAX phase was synthesized from TiC, Ti, and Al powders at 1380 °C under a constant argon flow. The washed, dried, and sieved Ti_3_AlC_2_ precursor was etched in a mixture of HCl and HF to produce multilayered MXene, which was then delaminated by dispersing the multilayer MXene in a LiCl solution to obtain single- and few-layer Ti_3_C_2_T_*x*_ MXene. A concentrated stock aqueous suspension was stored in the refrigerator in a sealed bottle under argon, and fresh aliquots were drawn on the day of the experiments.

### MXene Film Preparation

The samples were deposited on microstructured Si wafers (Irubis GmbH), N-doped Si wafers (Siegert Wafer GmbH), and Si wafers with 280 nm SiO_2_ layer (UniversityWafer, Inc.) for infrared, XRD, and resistivity measurements, respectively. 1 mg/mL aqueous suspension of Ti_3_C_2_T_*x*_ was prepared for each case and 50–100 μL was pipetted onto the substrates. This resulted in round films about 8 and 10 mm in diameter for infrared and XRD measurements, respectively. For resistivity measurements, the substrate was additionally plasma-cleaned to increase the hydrophilicity of the substrate. As a result, the MXene film covered the whole 10 × 10 mm^2^ substrate. Film thicknesses of about 1 to 2 μm were measured with a profilometer.

### Cation Exchange

The delamination step resulted in residual Li^+^ being present in the as-received sample. Cation exchange was performed on the drop-cast film to preserve the morphology of the film and achieve most comparable results across the measurement sets. 3 M solutions of chloride salts were pipetted onto the Ti_3_C_2_T_*x*_ film such that the whole film was covered (ca. 135 μL) and left to equilibrate for 1 h. This process was repeated a second time. Then, the film was rinsed thoroughly with water and allowed to dry before mounting into the humidity cell. In the case of Ti_3_C_2_T_*x*_ without intercalated cations, referred to as pristine Ti_3_C_2_T_*x*_, 1 M HCl was used to extract Li^+^ instead of the 3 M chloride salt.

### FTIR Measurements

Infrared spectra were collected in the attenuated total reflectance (ATR) mode with a Bruker 70v spectrometer using a room-temperature deuterated l-alanine-doped triglycine sulfate (DLaTGS) detector. The optical accessory was designed and built in-house to accommodate the microstructured Si wafer as the ATR element and provided an angle of incidence of 28.74°. Spectra were recorded from 6000 to 350 cm^–1^, thus covering simultaneously both the stretching and bending modes of water. The spectrometer was operated under vacuum (ca. 1 mbar) while the sample was inside a liquid cell at ca. 1 bar pressure. Each spectrum consisted of 64 scans and took approximately 1 min to record. Five spectra were recorded at each humidity and averaged to yield the final spectrum. Linear baselines were subtracted from the spectra prior to data analysis. An RH200 humidity generator (L&C Science and Technology) controlled with LabView was used to set the humidity. A combined temperature, pressure, and humidity sensor was placed inside the cell very close to the sample. The temperature and pressure remained stable throughout the humidity cycling at 27 °C and ca. 1 bar, respectively. The humidity was first set to 0% RH for 1.5 h to dry the MXene film. Note that these conditions are not sufficient to remove all intercalated water. Then, the humidity was increased at 5 percentage point increments up to 80% RH, after which the cycle was reversed, and the same steps followed back down to 0% RH. Each humidity step was maintained for 20 min prior to recording 5 FTIR spectra to ensure the spectra were stable.

### XRD Measurements

XRD patterns were collected with a Bruker D8 Advance X-ray diffractometer in the Bragg–Brentano geometry using Cu K_α_ radiation (λ = 0.154 nm) and a humidity cell built in-house equipped with a Kapton window. The data was collected in a 2θ range of 4–12.5° with a step size of 0.03° and 1.5 s collection time per step. The humidity was controlled with the same humidity generator as for the infrared measurements, with the following differences: The temperature inside the cell increased slightly during the measurement and ranged from 25 to 30 °C. It took a maximum of 15 min for the humidity to stabilize inside the cell. Each humidity step was maintained for 30 min, and the XRD patterns were collected every 10 min. Only patterns collected during a stable humidity reading were used. A reference measurement at 10% RH intervals was performed with a clean Si wafer inside the humidity cell to see the contribution from the Kapton window in the angle range of interest. The patterns were background-corrected and the *k*_α2_ contribution was removed with Bruker’s diffrac.EVA software. The 002 peaks were fitted with Voigt functions or with a Gaussian when the Lorentzian contribution was found to be negligible.

### Four-Point Probe Measurements

The resistance was measured continuously with a Keithley digital multimeter and a homemade four-point probe placed inside an HCP 50 humidity chamber (Memmert). The temperature was kept constant at 35 °C. The humidity was first set to 20% RH, the lowest value possible with the chamber, and the sample was allowed to equilibrate for an hour. Then, the humidity was increased at 5 percentage point increments up to 90% RH, after which the cycle was reversed, and the same steps followed back down to 20% RH. Each humidity step was maintained for 45 min, and the resistance value was averaged over the final 10 min for each humidity. Resistivity was calculated from the resistance values using the film thickness.

## Results and Discussion

### Hydration Shell of Intercalated Cations

**Figure 1:**
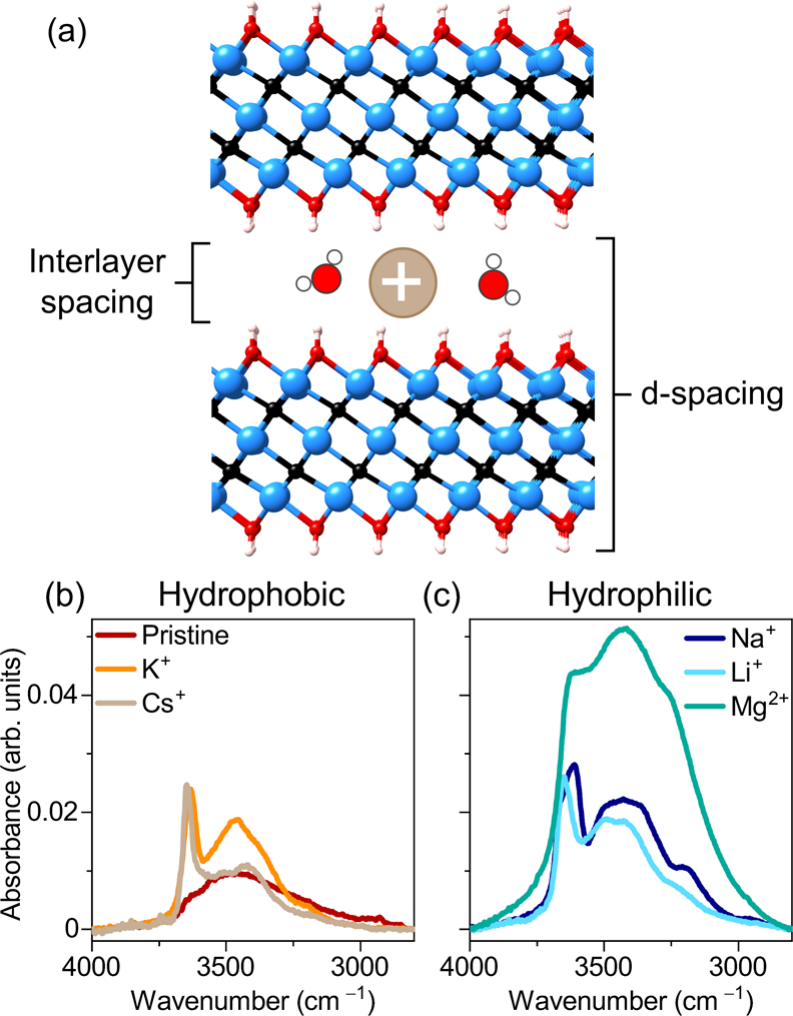
(a) Schematic structure of OH-terminated Ti_3_C_2_ with intercalated cations (light brown spheres) and water molecules. Ti atoms are shown in light blue, C in black, O in red, and H in white. (b, c) FTIR spectra of Ti_3_C_2_T_*x*_ films intercalated with different cations at 0% relative humidity in the O–H stretch region. (b) Pristine sample, K- and Cs-Ti_3_C_2_T_*x*_; (c) Na-, Li-, and Mg-Ti_3_C_2_T_*x*_.

Figure 1(a) shows the idealized structure of cation-intercalated OH-terminated Ti_3_C_2_ at low humidity. FTIR spectra in the O–H stretching mode region of the cation-intercalated Ti_3_C_2_T_*x*_ films at 0% RH are presented in Figure 1(b,c) (see the SI for spectra in the bending mode region). The samples are divided into two groups based on the hydration enthalpies of the cations (see Table S1 for values). The pristine Ti_3_C_2_T_*x*_ sample is grouped with “hydrophobic” K^+^ and Cs^+^ due to the very low intensity in the stretch region. The feature is very broad, and there is only a very weak sharp peak at 3650 cm^–1^. This indicates that the acid treatment removed most of the Li^+^ and that the amount of intercalated water is very low in the absence of an intercalating cation under these conditions. It has been reported previously^38^ that processing Ti_3_C_2_T_*x*_ in an acidic solution removes much of the residual Li^+^ cations that are intercalated into the Ti_3_C_2_T_*x*_ during delamination. Absorption at the lower-wavenumber tail of the O–H stretch region stems from water molecules in the hydration shell of hydrated protons that experience very strong H-bonding.^21^ The very low absorbance intensity compared to our previous report indicates that most hydrated protons are also removed during the rinsing process. When K^+^ are intercalated into the MXene film, the O–H stretch region becomes much narrower in shape, with greatly reduced intensity at lower wavenumbers, indicating that the water in the K^+^ solvation shell experiences weaker H-bonding. The sharp free O–H peak, on the other hand, is very intense and narrow. Cs-Ti_3_C_2_T_*x*_ exhibits very weak intensity at low humidity (on par with that of pristine Ti_3_C_2_T_*x*_), and an intense, narrow free O–H peak (similar to K-Ti_3_C_2_T_*x*_).

The “hydrophilic” cations Li^+^, Na^+^, and Mg^2+^ are shown in Figure 1c. Compared to pristine Ti_3_C_2_T_*x*_, Li-Ti_3_C_2_T_*x*_ shows much more spectral intensity in this region, indicating that there is more water intercalated between the MXene sheets. The free O–H peak at 3650 cm^–1^ is present, but is considerably weaker in intensity compared to K^+^ and Cs^+^. The shape of the O–H stretch envelope is also broader than that found for K^+^ and Cs^+^, with more intensity at lower wavenumbers. When Li^+^ is exchanged with Na^+^, a very similar spectral intensity is seen at all wavenumbers in the O–H stretch region. Mg-Ti_3_C_2_T_*x*_ exhibits a very high spectral intensity in this region.

In a system with one H_2_O molecule and one metal cation, the electrostatic interaction between the positively charged cation and the dipole moment of the water molecule causes a red shift in the symmetric and asymmetric O–H stretching modes of H_2_O.^39,40^ When more water molecules are added, water–water interactions begin to compete with water–ion interactions. For higher-charge-density cations, such as Li^+^ and Mg^2+^, water–ion interactions are initially stronger than for, e.g., K^+^ and Cs^+^ that have much lower charge density. It has been observed that already the third water molecule added to a M^+^(H_2_O)_n_ (where M = K^+^ or Cs^+^) cluster has such weak water–ion interaction that it prefers to form hydrogen bonds with the existing water molecules instead of binding directly with the cation.^41,42^ For Li^+^ and even Na^+^, this does not happen until the fourth added H_2_O. At the same time, the charge density of the cation will polarize water molecules in the primary hydration shell. The higher the charge density, the higher the degree of polarization and the stronger the H-bond formed by this water and a second water molecule in a secondary hydration shell.^41^

Spectral intensity below about 3600 cm^–1^, even at the lowest humidity, indicates that the intercalated water in all samples takes part in H-bonding. This means that either we are not observing small M^+^(H_2_O)_n_ clusters where *n* < 3, or the H_2_O molecules H-bond with surface groups on the MXene layers, or H-bonded hydroxyl terminations contribute significantly to the spectrum. The broadness of the stretches of the O–H stretch envelope suggests a large number of H-bonding configurations and points to the presence of larger M^+^(H_2_O)_n_ clusters. Without an additional driving force, such as high temperature or low pressure, it is impossible to remove all of the water from the cation hydration shell.^25^

Non-H-bonded hydroxyl terminations on TiO_2_ under UHV conditions were observed at ca. 3660 cm^–1^,^43^ whereas H-bonded hydroxyl groups under atmospheric conditions were found to undergo a considerable red shift to 3279 cm^–1^.^44^ Since there is abundant H-bonding occurring for all MXene samples, non-H-bonded −OH surface groups are very unlikely to contribute to the sharp peak at 3650 cm^–1^. The near absence of this peak in the pristine sample supports the conclusion that Li^+^ has been largely removed and that this peak originates from the free O–H stretch of water molecules in the cation hydration shell rather than surface hydroxyl groups.^21^

The smaller hydration energies of K^+^ and Cs^+^ compared to Li^+^, Na^+^ and Mg^2+^ mean that water molecules in the solvation shell are less strongly bound to the cation and removed more easily. Experimentally determined numbers of tightly bound water molecules for these cations were zero for K^+^ and increased in the order Na^+^ (0.22) < Li^+^ (0.6) < H^+^ (1.9) < Mg^2+^ (5.8).^45^ The smaller amount of water remaining in the interlayer space, combined with the larger size of the bare cations that reduces the H-bonding strength of the remaining water molecules, contributes to the strong free O–H peak observed for “hydrophobic” K- and Cs-Ti_3_C_2_T_*x*_. On the other hand, stronger H-bonding is present with the “hydrophilic” cations Mg^2+^, Li^+^, and Na^+^.^12^ The broad and intense O–H band in the FTIR spectrum of Mg-Ti_3_C_2_T_*x*_ at 0% RH reflects the high number of tightly bound water molecules for Mg^2+^, which is very close to a full first hydration shell.^46^ H^+^ is considered a strongly hydrated species,^12^ which is reflected in the strong H-bonding, but not in the low intensity, observed in the FTIR spectrum. This may be related to the small coordination number (2–3) of H^+^ compared to other cations.^47^

### Building Up a Confined Water Layer with Humidity

**Figure 2:**
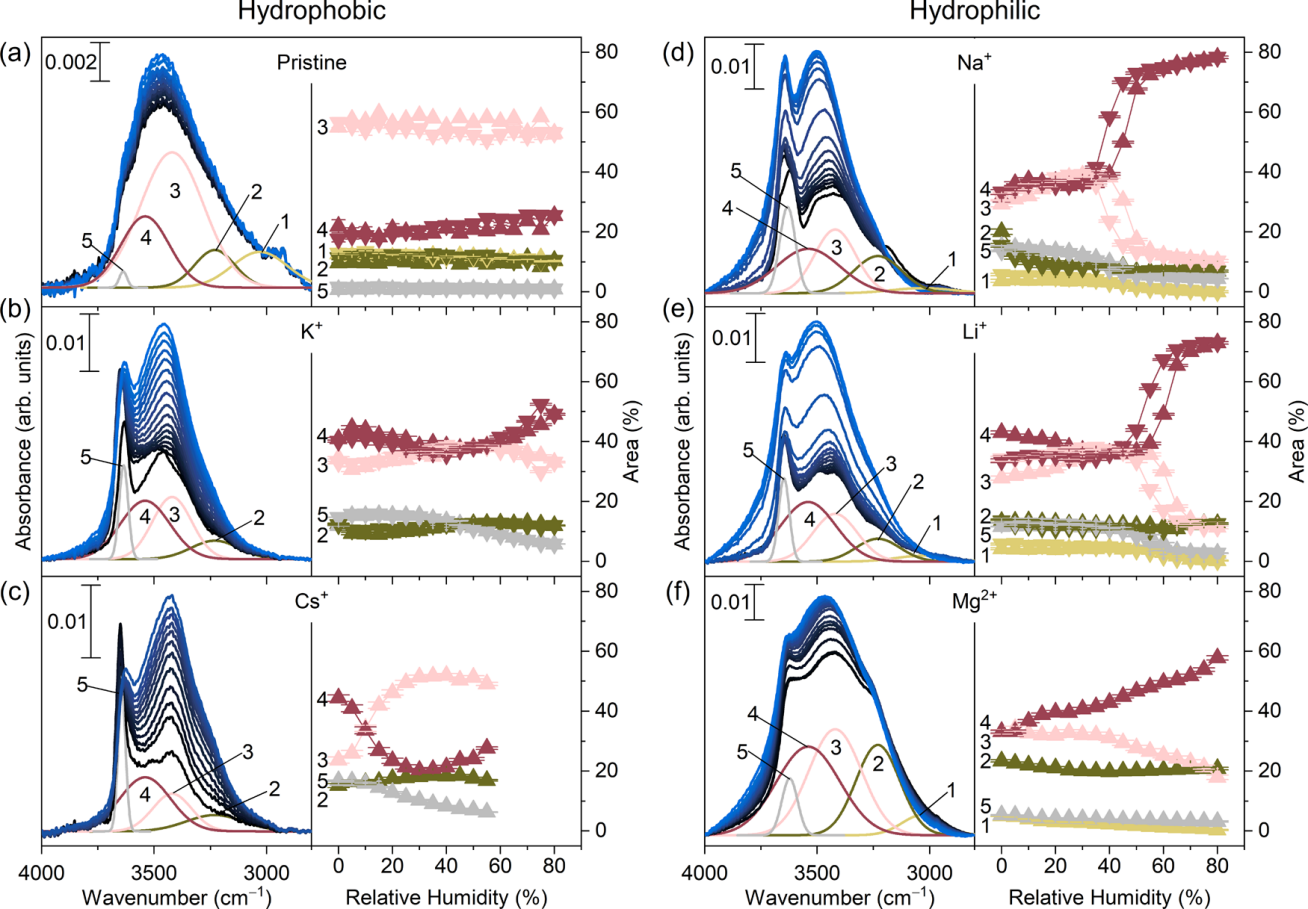
FTIR spectra in the O–H stretching mode region as a function of humidity. The color scheme varies from black at 0% RH to blue at 80% RH. Peak fit of experimental data recorded at 0% RH (peak 1: yellow; peak 2: green; peak 3: pink; peak 4: purple; peak 5: gray). Integrated peak areas from the full hydration–dehydration cycle. For Cs-Ti_3_C_2_T_*x*_ and Mg-Ti_3_C_2_T_*x*_, only a partial cycle is shown due to the condensation of water in the humidity cell during measurement.

The FTIR spectra in the O–H stretching mode region during hydration are shown in Figure 2. To gain more insight into how the cation identity influences the hydrogen-bonding state of the intercalated water in Ti_3_C_2_T_*x*_, the FTIR spectra were fitted with five Gaussian peaks, following previous studies.^48−50^ In general, the frequency of the O–H stretch reports on the H-bonding state of a water molecule with lower frequencies indicating stronger H-bonding. The O–H stretching region is not straightforward to fit due to the broad nature of the peaks. In order to better compare trends across the samples and during hydration–dehydration cycles, the widths of the peaks were constrained and the frequencies were fixed for all peaks except for the free O–H peak, which is narrow and separated from the main broad bands. The peak areas are reported as percentage fractions of the total area.

Striking differences in the spectral shape and intensity are observed between the different samples. The pristine Ti_3_C_2_T_*x*_ MXene sample in Figure 2a presents very low intensity at all humidity values, despite the large hydration enthalpy of protons. A significant part of the integrated area is attributable to low-frequency modes at 3030 and 3230 cm^–1^ (peaks 1 and 2), suggesting strong hydrogen bonding of the water, consistent with water molecules hydrating residual protons within the film. Peaks 3 and 4, located at 3420 and 3540 cm^–1^, correspond to water molecules participating in moderate and weak hydrogen bonding, respectively. The area of peak 3 for pristine Ti_3_C_2_T_*x*_ is larger than that seen in other samples due to stronger H-bonding attributed to residual protons residing in the interlayer space. K-Ti_3_C_2_T_*x*_ (Figure 2b) shows a steady increase in intensity, with no stepwise increase. The sharp peak at 3650 cm^–1^ (peak 5) becomes less intense with increasing humidity, consistent with its assignment to free O–H that decreases as more water enters the interlayer space. The water present in the K-intercalated sample exhibits weak hydrogen bonding, indicated by the lack of intensity at 3030 cm^–1^ and strong free O–H stretching peak at 3650 cm^–1^. Similar to K^+^, Cs-intercalated Ti_3_C_2_T_*x*_ in Figure 2c shows only a small amount of water with no intensity at 3030 cm^–1^ and a relatively large area attributed to free O–H stretching, indicative of weak H-bonding. Very little increase in the spectral intensity is observed for Cs-Ti_3_C_2_T_*x*_ until condensation inside the humidity cell from ca. 60% RH introduced an overlapping signal of bulk water into the spectra. Spectra recorded at relative humidity >55% are therefore omitted from Figure 2(c). For both K-Ti_3_C_2_T_*x*_ and Cs-Ti_3_C_2_T_*x*_, the increase in humidity causes an initial increase and decrease in the areas of peaks 3 (3420 cm^–1^) and 4 (3540 cm^–1^), respectively. At higher humidity, the trend is reversed, and the area of peak 4 begins to increase.

In the case of Li-Ti_3_C_2_T_*x*_, the spectral intensity initially increases slowly and then presents a sudden stepwise increase at around 55% RH before slowing down again (Figure 2e. When Li^+^ is exchanged with Na^+^, a similar behavior is observed, as seen in Figure 2d. At low humidity, very similar spectral intensity is seen in the spectra, and a sharp free O–H peak is prominent. Mg-Ti_3_C_2_T_*x*_ (Figure 2f) exhibits very high spectral intensity at low humidity, with a sudden increase at 10% RH. After that, very little increase is observed. The free O–H peak is much less prominent in this sample due to the strong overall intensity in the O–H stretch region. Condensation inside the humidity cell occurred at 80% RH, affecting the spectra during dehydration.

All cations show an increase in peak 3 and a decrease in peak 4 at low humidity, pointing to stronger H-bond formation in the low-humidity regime. At moderate humidity levels, this behavior is reversed: we now see peak 4 increasing and peak 3 decreasing. For the chaotropic K^+^ and Cs^+^, the peak evolution is not so drastic, but Li-Ti_3_C_2_T_*x*_, Na-Ti_3_C_2_T_*x*_, and Mg-Ti_3_C_2_T_*x*_ manifest a sudden, steep increase and decrease of the relative areas of peaks 4 and 3, respectively. This sudden increase suggests additional water in a second hydration shell or a second water layer, which experiences weak hydrogen bonding due to confinement.

Calculations have been performed on Ti_3_C_2_T_*x*_-metal cation systems in the fully hydrated form to elucidate the position of different cations within the interlayer space.^27,34^ Osti et al. determined that Li^+^ sits between the MXene surface and the water layer and bonds tetrahedrally with two hydroxyl groups and two water molecules, whereas K^+^ ions position themselves in the center of the water layer and form a planar hydration structure. Na^+^ behavior was in between these two extremes.^34^ Gao et al.,^27^ however, concluded that Li^+^, Na^+^, and K^+^ were positioned between the MXene surface and the water, but at different distances, and only Cs^+^ and Mg^2+^ stayed away from the MXene surface. At this stage, the role of the ion distance to the MXene plane on the ion hydration profile is not obvious and may rather alter the MXene surface chemistry due to M-O bonding with the surface groups.^51^

### Local Acidification due to Mg^2+^ Intercalation

**Figure 3:**
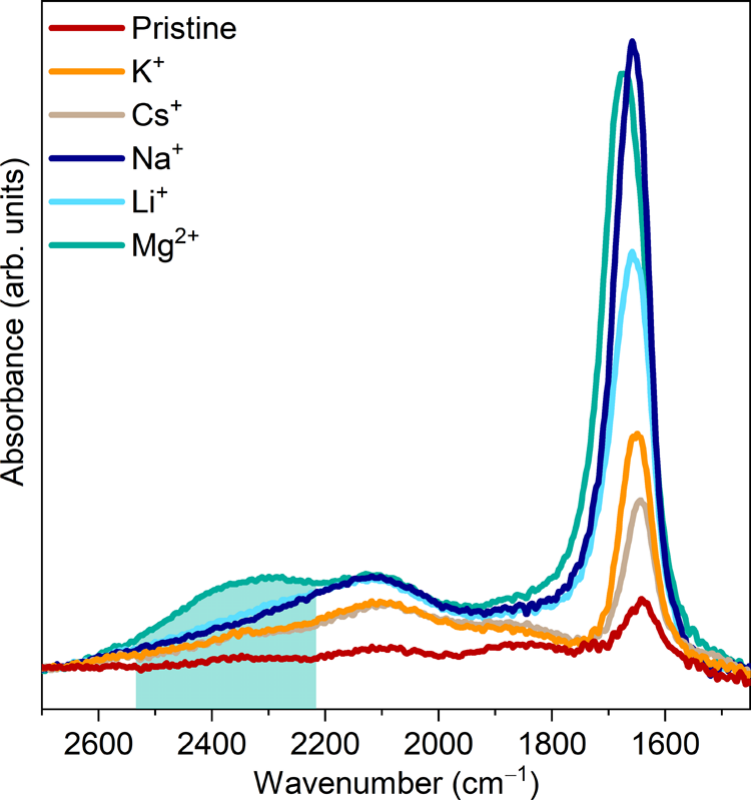
FTIR spectra of MXene with different intercalants at 80% RH (55% RH for Cs-Ti_3_C_2_T_*x*_) in the range 2700–1450 cm^–1^.

Mg-Ti_3_C_2_T_*x*_ displays an additional vibrational feature at around 2400 cm^–1^ (Figure 3) which may arise from hydrated protons within the MXene interlayer as previously reported by us.^21^ Mg^2+^ is known to act as a Lewis acid and form charge transfer complexes with a solvating water molecule,^52^ leading to water fragmentation as described by the following reaction
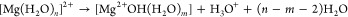


The protons generated during this reaction can then intercalate into the MXene film together with Mg^2+^. Electrochemical measurements reported in ref (28) indicate an additional pseudocapacitive process in MgSO_4_ electrolyte, similar to that observed in acidic electrolyte, supporting the hypothesis of proton intercalation in this sample. Interestingly, this phenomenon is not observed with any of the monovalent cations.

### Modulation of Ti_3_C_2_T_*x*_ MXene Interlayer Spacing

**Figure 4:**
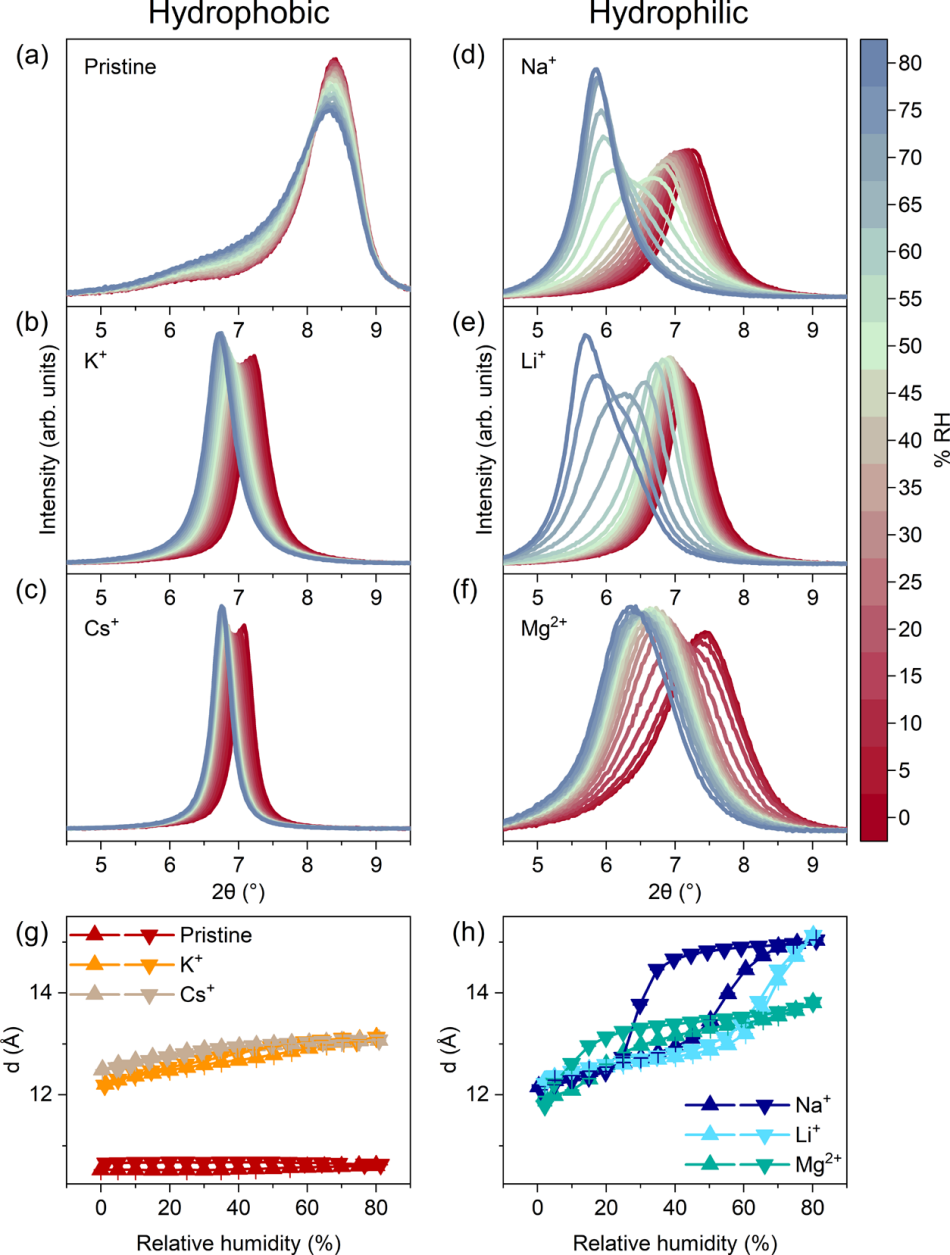
*In situ* XRD as a function of humidity for Ti_3_C_2_T_*x*_ films intercalated with different cations. (a-f) XRD patterns in the 002 region during hydration. The color scheme varies from red at 0% RH to blue at 80% RH. (g, h) The *d*-spacing extracted from patterns recorded during a full hydration–dehydration cycle and plotted as a function of humidity.

In Figure 4, the XRD patterns in the range covering the 002 peak are presented. From the 002 peak, the *d*-spacing can be calculated based on Bragg’s law, and the interlayer spacing is estimated by comparison with a dry multilayered Ti_3_C_2_T_*x*_ powder, the *d*-spacing of which has been reported to be 9.4 Å.^33^ There is a gap of about 2.7 Å between two Ti_3_C_2_T_*x*_ sheets with minor variations depending on the strength of repulsive and attractive forces between the terminations as well as the magnitude of stacking disorder.^53,54^ Pristine Ti_3_C_2_T_*x*_, i.e., acid-treated Ti_3_C_2_T_*x*_, at 0% RH shows a main peak at 8.4° with some minor contributions at lower angles. The main peak position corresponds to a *d*-spacing of 10.5 Å and an interlayer spacing of 1.1 Å. Considering that the size of a water molecule is about 2.75 Å and that he calculated *d*-spacing for a proton-intercalated Ti_3_C_2_O_2_ with one water layer is reported to be 12.5 Å,^32^ this value suggests that there is no water confined in the interlayer space. The FTIR data, however, prove that there is some water and protons present in the sample, although this could be trapped in pockets within the film rather than confined between the MXene sheets. If there is one confined water layer, the smaller *d*-spacing could be due to the surface terminations holding the sheets closer together via electrostatic interactions and H-bonding. The modeling in^32^ was performed with uniform -O terminations, but in a real sample -F and -OH terminations will also be present, and the surface terminations are known to influence the *d*-spacing.^54^ The 002 peak shifts by 0.1° as the humidity is increased to 80% RH. This translates to a negligible increase of 0.1 Å in the interlayer spacing, indicating that very little water intercalates between the Ti_3_C_2_T_*x*_ sheets even at high humidity. In this case, the lack of water entering the interlayer space supports the conclusion of zero confined water layers, as a complete removal of intercalated water in Ti_3_C_2_T_*x*_ is usually irreversible.^54^

In K-intercalated Ti_3_C_2_T_*x*_, the 002 peak is centered at 7.3° (12.2 Å *d*-spacing, 2.8 Å interlayer spacing) at 0% RH, and shifts downward linearly throughout the humidity range. At 80% RH, the peak is positioned at 6.7°, translating to a *d*-spacing of 13.1 Å and an interlayer spacing of 3.7 Å. This suggests that even at high humidity, there is only one layer of water in K-Ti_3_C_2_T_*x*_. Another interesting point is that the 002 peak is very narrow, indicating regular interlayer spacing throughout the sample. When Cs^+^ is intercalated between the Ti_3_C_2_T_*x*_ sheets, the 002 peak is very similar to that of K-Ti_3_C_2_T_*x*_ in its sharp, narrow profile and its position as a function of humidity. The peak position starts out at 7.1° and shifts gradually to 6.8° as the humidity increases to 80% RH. This corresponds to a *d*-spacing that increases from 12.5 to 13.1 Å and interlayer spacing that increases from 3.1 to 3.7 Å.

In contrast, Li-Ti_3_C_2_T_*x*_ presents a 002 peak at 7.2° at 0% RH, corresponding to a *d*-spacing of 12.3 Å and an interlayer spacing of 2.9 Å, which are very close to the size of a water molecule. This indicates that a monolayer of water intercalated between the Ti_3_C_2_T_*x*_ sheets. As the humidity increases, the angle initially shifts downward almost linearly until about 60% RH when there is a distinct step in the peak position to lower angles. At 80% RH, the highest humidity measured here, the 002 peak is positioned at 5.7°, translating to a *d*-spacing of 15.5 Å and an interlayer spacing of 6.1 Å. This is considerably larger than what is observed with K^+^ and Cs^+^ and suggests that at high humidity there are two layers of water in Li-Ti_3_C_2_T_*x*_ rather than just one layer as seen with K^+^ and Cs^+^.

When Li^+^ is exchanged with Na^+^, a very similar behavior is observed. At 0% RH, the 002 peak is centered at 7.3° (12.2 Å *d*-spacing and 2.8 Å interlayer spacing). As the humidity increases, the peak position again shifts downward in a linear manner until a sudden decrease is observed, this time around 40% RH. At 80% RH, the 002 peak is positioned at 5.9°, translating to a *d*-spacing of 15.1 Å and an interlayer spacing of 5.7 Å. Li-Ti_3_C_2_T_*x*_ and Na-Ti_3_C_2_T_*x*_ therefore start out and end up at very similar *d*-spacings and both exhibit a discontinuous increase in the *d*-spacing as a function of humidity. The main difference is that this stepwise increase in the *d*-spacing occurs at a lower humidity in Na-Ti_3_C_2_T_*x*_ compared to Li-Ti_3_C_2_T_*x*_.

Lastly, Mg^2+^ was intercalated into the Ti_3_C_2_T_*x*_. At 0% RH, the 002 peak position was found at 7.4° (12.0 Å *d*-spacing, 2.6 Å interlayer spacing), similar to those of all cation-intercalated samples in this study. Again, a stepwise sudden shift to lower angles was observed in the XRD pattern but at much lower humidity (ca. 15% RH) than what was observed for Li-Ti_3_C_2_T_*x*_ and Na-Ti_3_C_2_T_*x*_. At 80% RH, the 002 peak was positioned at 6.4° (13.8 Å *d*-spacing, 4.4 Å interlayer spacing). The interlayer spacing is somewhat smaller than expected for two water layers, as seen with Li^+^ and Na^+^, but considerably larger than that seen with K^+^ and Cs^+^ that only accommodate one water layer. This may be explained by a stronger electrostatic attraction between the positively charged cation and negatively charged MXene sheets due to the high charge density of the doubly charged Mg^2+^ ion.

In the presence of hydrophilic Na^+^, Li^+^, and Mg^2+^, the 002 diffraction peak is broader than that found in MXene intercalated with hydrophobic cations. In the transition region from one to two water layers, as the interlayer spacing increases to accommodate more water, the peak broadens further. Especially in the case of Na^+^ and Li^+^, heterogeneous hydration structures are clearly present, as also reported by Célérier et al. for Li-intercalated Ti_3_C_2_T_*x*_.^30^ The hysteresis observed in Na-intercalated Ti_3_C_2_T_*x*_ is in agreement with previously reported XRD data;^25^ however, the lack of hysteresis in the Li-intercalated sample is surprising and we are unable to provide a clear explanation for this.

Our observations with delaminated Ti_3_C_2_T_*x*_ are in agreement with previous studies of multilayered Ti_3_C_2_T_*x*_ with intercalated K^+^, Na^+^, Li^+^, and Mg^2+^ as a function of humidity.^25,30^ At low humidity, all three samples presented a 002 reflection corresponding to a structure with one water layer between the Ti_3_C_2_T_*x*_ sheets. Furthermore, all samples except for K-intercalated Ti_3_C_2_T_*x*_ displayed a discontinuous shift of the 002 reflection with an increase of ca. 3 Å in the interlayer spacing at higher humidities, attributed to a transition from one to two water layers in the interlayer space. We added Cs^+^, a hydrophobic cation with low charge density, to the list of intercalants. The observed behavior of Cs-Ti_3_C_2_T_*x*_ is almost identical to that of K-Ti_3_C_2_T_*x*_, another weakly hydrated cation. We have also measured a cation-free film for the first time as a function of humidity.

Previously reported values for fully hydrated, multilayered K-intercalated Ti_3_C_2_T_*x*_ and Mg-intercalated Ti_3_C_2_T_*x*_ were slightly smaller and larger, respectively, compared to what we found.^25,55^ This suggests that the delamination, as performed for our Ti_3_C_2_T_*x*_ samples, may not be the determining factor when it comes to the *d*-spacing; the surface terminations may be much more important in that regard. Both of the above studies used a different MAX phase and, crucially, a different etching and delamination procedure, all of which affect the nature and distribution of functional groups on the MXene surface. For instance, increasing HF etching time has been linked to a higher fluorine content and an increasing interlayer distance in multilayer Ti_3_C_2_T_*x*_ powders.^56^

### Influence of Confined Water Layer on the Resistivity of MXene Films

**Figure 5:**
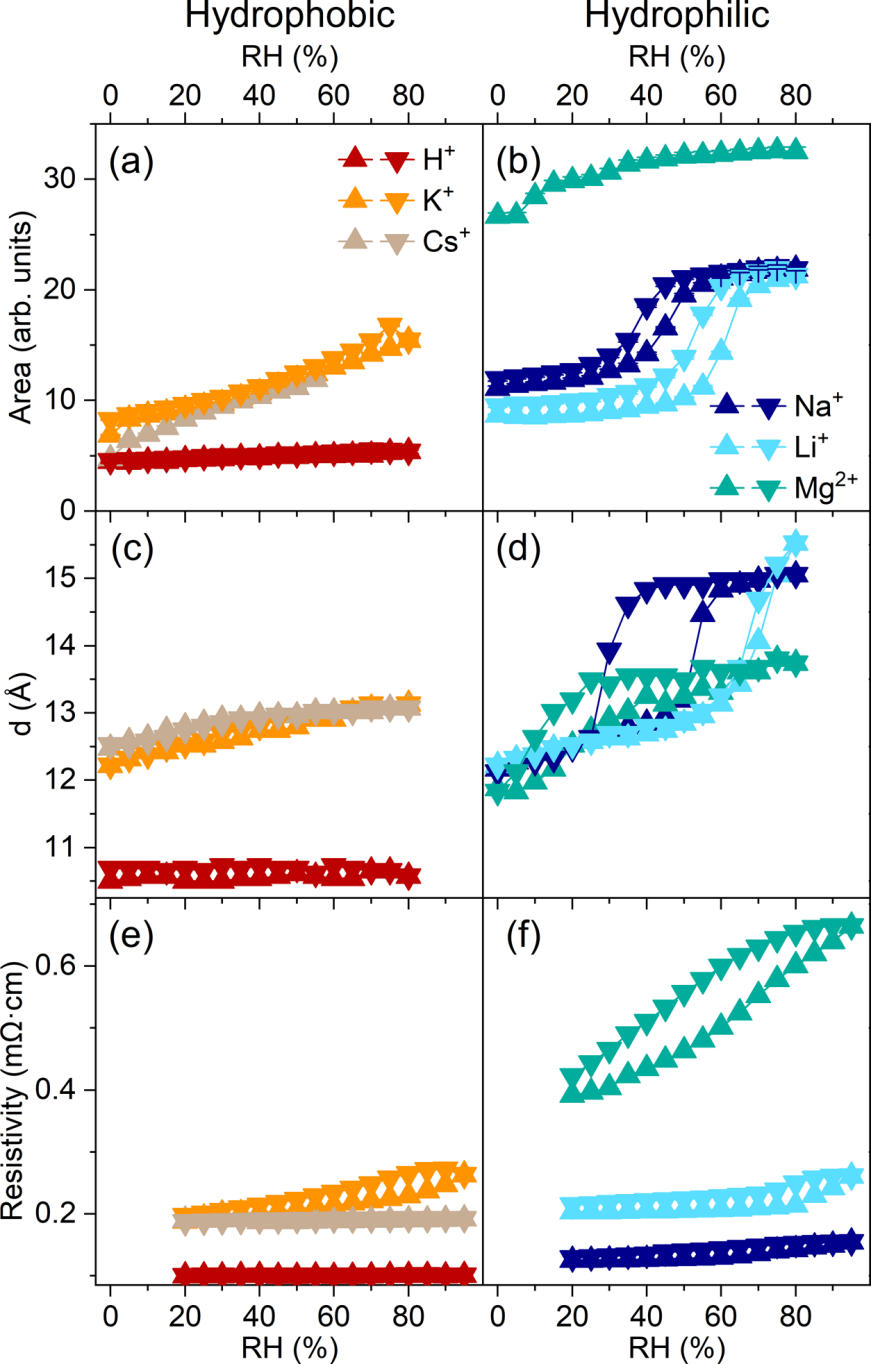
Effect of humidity on hydrogen bonding, interlayer spacing, and resistivity. (a, b) Integrated area of O–H stretching mode, (c, d) *d*-spacing calculated from XRD patterns, and (e, f) resistivity as a function of humidity for pristine, Li-, Na-, K-, Cs-, and Mg-Ti_3_C_2_T_*x*_ during a full hydration–dehydration cycle. In (a) and (b) for Cs- and Mg-Ti_3_C_2_T_*x*_, data points are only shown for hydration due to water condensation inside the cell.

Figure 5 shows how the total integrated areas of the O–H stretching mode and the water bending mode calculated from the FTIR measurements correlate with the *d*-spacing obtained from the XRD patterns and the resistivity. Clear trends are observed in the data. Pristine Ti_3_C_2_T_*x*_ and Mg-Ti_3_C_2_T_*x*_ have the lowest and highest resistivities, respectively, in agreement with the lowest and highest areas of the O–H stretch region for these two samples. The humidity dependence of K- and Cs-Ti_3_C_2_T_*x*_ is similar across all measurements. The data for Li-Ti_3_C_2_T_*x*_ show a discrete step in the area of the stretching mode region and a discrete step in the resistivity. Na-Ti_3_C_2_T_*x*_ also presents a clear step in the stretching mode area, and a less well-defined step in the resistance starting at lower % RH values than seen in Li-Ti_3_C_2_T_*x*_. Because resistivity measurements were not possible below 20% relative humidity, the same correlation for Mg-Ti_3_C_2_T_*x*_ cannot be made at this time. The XRD patterns and the evolution of the integrated area of the water stretch region of Na-intercalated Ti_3_C_2_T_*x*_ show that Na^+^, Li^+^, and Mg^2+^ can support two layers of water in the MXene interlayer space due to their higher hydration enthalpies.

Our results show that chaotropic, weakly hydrated cations (K^+^, Cs^+^) in 2D confinement between negatively charged, hydrophilic Ti_3_C_2_T_*x*_ surfaces influence water in a manner that is distinctly different from the behavior of kosmotropic cations (Li^+^, Na^+^, Mg^2+^). Chaotropic cations reduce the amount of confined water between MXene sheets and prevent the formation of a water bilayer within the structure, whereas kosmotropic cations show more abundant water present in the structure, with a bilayer formation at a relative humidity specific to the cation. At low humidity levels we see an increase in moderately strong H-bonding, and with higher humidity weaker H-bonding begins to dominate. This is attributed to the transition between mainly water-cation and mainly water–water interactions as RH increases. The kosmotropic cations show a sudden increase in weaker H-bonding that directly correlates with the abrupt expansion of the *d*-spacing, whereas the chaotropic cations show a smooth transition to weaker H-bonding, consistent with a minor and linear expansion of the *d*-spacing. The changes in conductivity seem to correlate more with the amount of water than the *d*-spacing, as we see much lower conductivity in Mg-Ti_3_C_2_T_*x*_ compared to other hydrophilic cations despite the similar *d*-spacing.

These results show the impact of cations on the structure and amount of water in the 2D confinement. In bulk solutions, hydrophobic cations K^+^ and Cs^+^ may be beneficial electrolyte additives to improve the kinetics of HER based on their weakly hydrated structure, while strongly hydrated Li^+^, Na^+^, and Mg^2+^ reduce water activity in aqueous batteries. However, since the kosmotropic Li^+^, Na^+^, and Mg^2+^ induce a larger water content between the MXene sheets, they could also increase the HER in strongly confined systems.

When the cations are confined between the Ti_3_C_2_T_*x*_ sheets, we add not only confinement effects to an already complicated picture of competing water–ion and water–water interactions but also MXene surface groups with the ability to H-bond with the intercalated water. Recording the spectral signature of the MXene film during dehydration under elevated temperature in vacuum could enable the observation of desorption of surface OH groups and partial or complete desolvation of the intercalated cation.

## Conclusions

We have presented here a multimodal *in situ* study of confined water in Ti_3_C_2_T_*x*_, intercalated with different cations (K^+^, Cs^+^, Na^+^, Li^+^, and Mg^2+^), as a function of humidity. The chosen techniques (FTIR, XRD, 4-point probe) complement each other by probing different aspects of the state of the confined water. Two different types of behavior were identified depending on the hydrophilic–hydrophobic nature of the cation. K^+^ and Cs^+^, two weakly hydrated cations, produce a very similar response to humidity: only a small amount of weakly H-bonded water is present at 0% RH, the *d*-spacing does not expand with humidity, and the conductivity remains high. Strongly hydrated cations Na^+^, Li^+^, and Mg^2+^ show more strongly H-bonded water at 0% RH, a sudden increase in the amount of water as determined by FTIR, an abrupt expansion in the *d*-spacing, and an increase in the resistivity. This work illustrates that the local microenvironment (e.g., water H-bonding and acidity) in the MXene interlayer may be tuned by changing the intercalated cation. The cation also strongly influences the properties of MXene, such as the electrical conductivity of films.

## Supporting Information Available

Cation hydration enthalpies and FTIR spectra in the water bending mode region (PDF)
