# Supplementary material for: Tuning the Microenvironment of Water Confined in Ti3C2Tx MXene by Cation Intercalation
Source: J Phys Chem C Nanomater Interfaces. 2024 Feb 14;128(7):2803–13. doi: 10.1021/acs.jpcc.4c00247 (PMC10895661; doi:10.1021/acs.jpcc.4c00247)
Supplement: Supplementary file 1 — jp4c00247_si_001.pdf [file jp4c00247_si_001.pdf]

# Tuning the Microenvironment of Water Confined in $Ti_3C_2T_x$ MXene by Cation Intercalation – Supporting Information

Mailis Lounasvuori*[a], Teng Zhang[b], Yury Gogotsi[b], Tristan Petit[a]

[a] Helmholtz-Zentrum Berlin für Materialien und Energy GmbH, Nanoscale Solid-Liquid Interfaces, Albert-Einstein-Str. 15, 12489 Berlin, Germany

[b] A.J. Drexel Nanomaterials Institute and Department of Materials Science and Engineering, Drexel University, Philadelphia, Pennsylvania 19104, United States

*Corresponding author

**Table S1.** Experimental hydration enthalpies of selected cations.[1]

| Ion | $-\Delta H^{\circ}_{hyd}$ |
|---|---|
| $H^+$ | 1091 |
| $Li^+$ | 519 |
| $Na^+$ | 409 |
| $K^+$ | 322 |
| $Cs^+$ | 264 |
| $Mg^{2+}$ | 1921 |

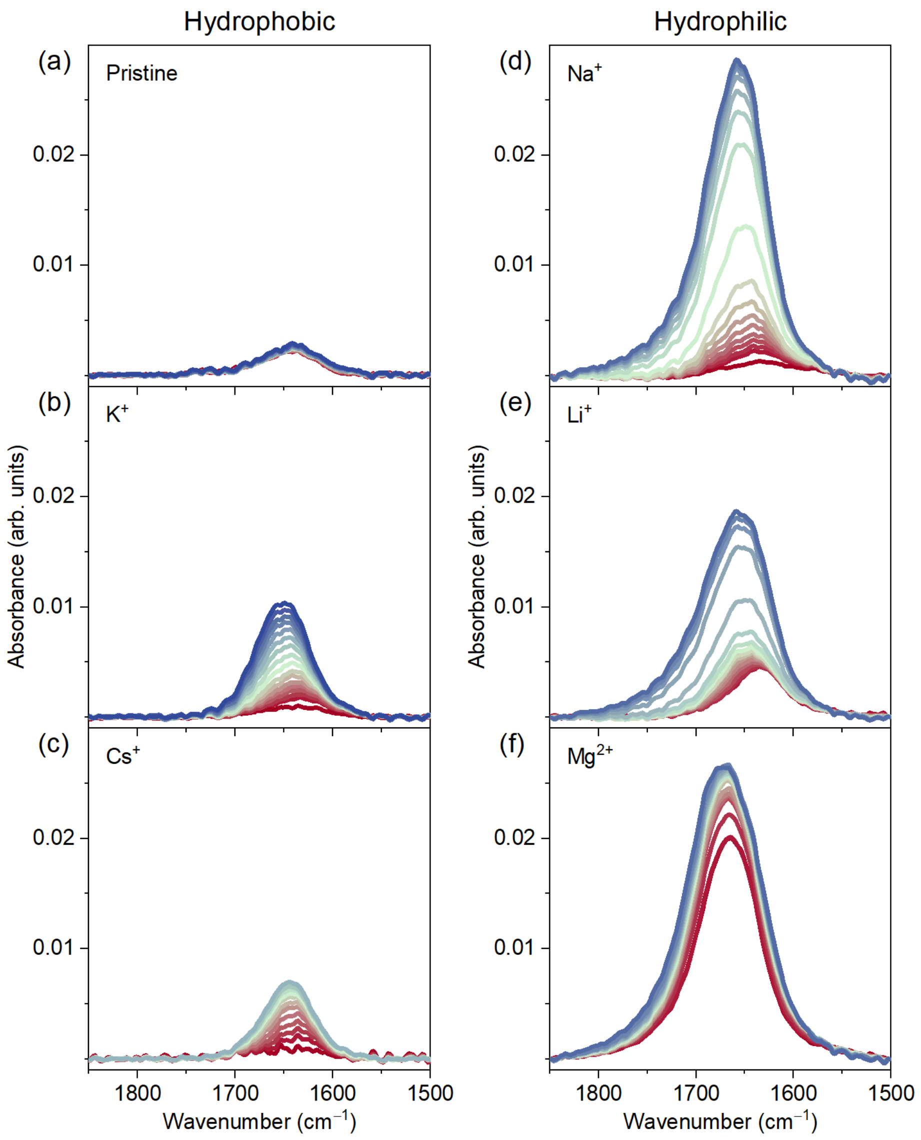


**Figure S1.** FTIR spectra in the water bending mode region as a function of humidity. The color scheme varies from red at 0% RH to blue at 80% RH.

FTIR spectra in the water bending mode region as a function of humidity are presented in Figure S1. They follow the same pattern as the O-H stretching mode: the pristine sample and hydrophobic cations induce only weak intensity at all humidity values, whereas a stepwise increase in the intensity is observed for the hydrophilic cations.

The bending mode of water is narrower than the stretching mode and generally fitted with a maximum of two components[2,3] as opposed to 5-6 components for the stretching mode.[4–6] Despite the smaller number of distinct components attributed to different H-bonding states, the frequency of the bending mode is also sensitive to the H-bonding strength, appearing at 1595 $cm^{-1}$, 1645 $cm^{-1}$ and 1735 $cm^{-1}$ in the gas, liquid and solid phases, respectively.[7,8] The bending mode therefore shifts to higher frequencies with increasing H-bonding strength, unlike the stretching mode where a redshift is associated with stronger H-bonds. A linear

relationship for this anti-correlation has been formulated by Falk based on experimental observations:[9]

$$\nu_2 = 1590.6 + 0.2583(3706 - \nu_{OH}) \qquad \text{Eq. S1}$$

where $\nu_2$ is the bending mode and $\nu_{OH}$ the stretching mode. The bending mode region was fitted with one Gaussian peak to determine how the center frequency evolves with humidity. In Figure S2, the experimental frequencies are compared with the calculated frequencies obtained using Equation S1, where $\nu_{OH}$ is the weighted average of the five fitted components of the O-H stretching region.

For all samples except pristine $Ti_3C_2T_x$ and Mg-$Ti_3C_2T_x$, the frequency is low at low humidity and shifts to higher frequency as the humidity increases. Due to the negligible signal intensity, no shift is observed in pristine $Ti_3C_2T_x$, although the area does increase about 20% from low to high humidity. Similarly, the calculated frequency remains nearly constant. When $Mg^{2+}$ is intercalated into the sample, the bending mode frequency is significantly higher, reflecting the larger amount of water present. In general, weakly hydrated cations and the pristine sample display experimental bend frequencies lower than the calculated value throughout the humidity range, whereas strongly hydrated cations induce a lower than expected frequency of the water bending mode at low humidity and a higher experimental value at high humidity. The transition occurs at the humidity at which a transition to a bilayer of water is observed in XRD. Bending mode frequencies lower than that predicted by Equation S1 have been attributed to cation-water interactions[9] and similarly low frequencies at low humidity have been reported previously for water in montmorillonite clays that also host cations and layers of water between individual sheets.[10]

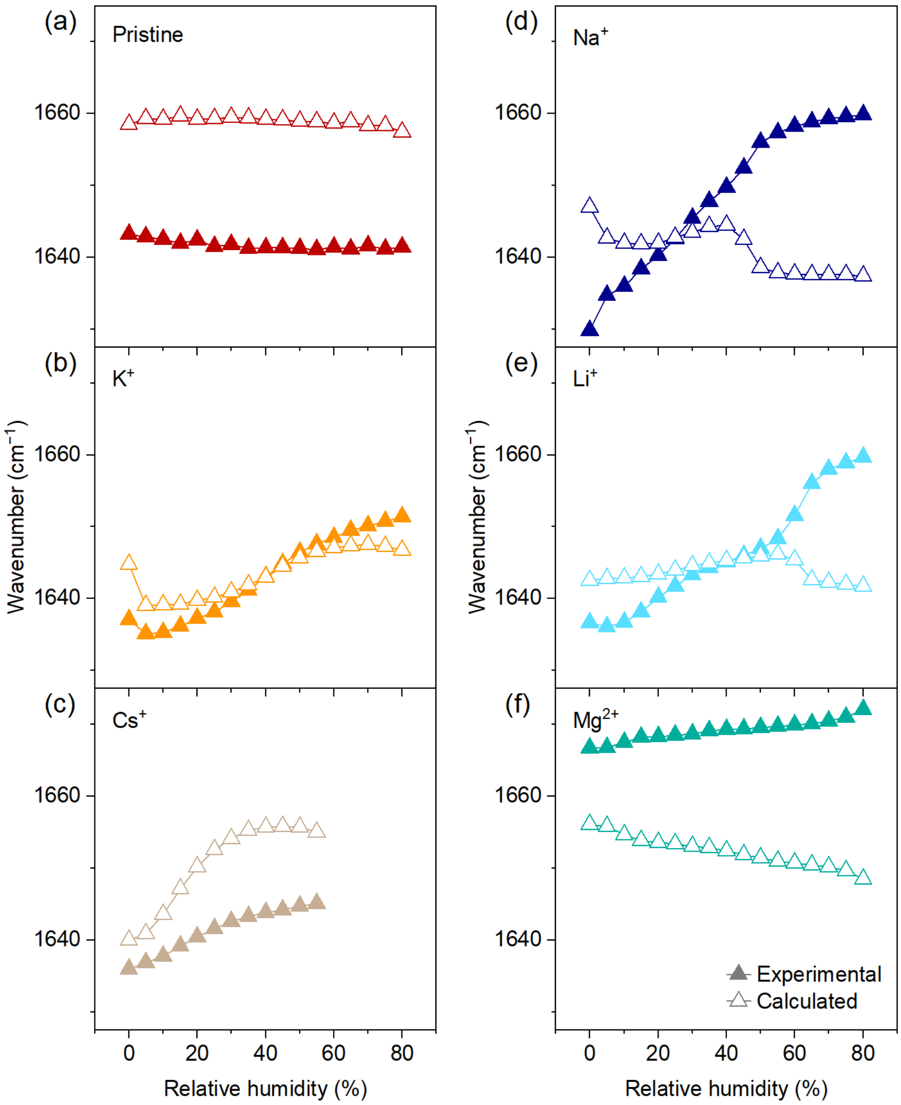


**Figure S2.** Experimental peak frequencies of the water bending mode Gaussian compared to the calculated bending mode frequencies estimated from the experimentally found O-H stretching mode frequencies.

Quantification of water intercalated into a MXene film is challenging, because the dipole moment of water is strongly enhanced by H-bonding and the relative intensities of O-H stretches participating in different H-bonding configurations can therefore differ significantly.[11] Ion-water interactions will also influence the dipole moment and thereby the intensity of absorption by the water molecule.[12] The stretching mode of water has the added disadvantage of overlapping with other O-H stretches, such as hydroxyl groups on the MXene surface. Despite these issues, by carefully controlling the reproducibility of MXene film preparation and monitoring both the stretch and bend regions, we can compare the relative amounts of water in each sample.

The integrated area of the bending mode for Na-$Ti_3C_2T_x$ is much smaller than that of Li-$Ti_3C_2T_x$ at 0% RH, even though the stretching mode areas are comparable. The intensity of

the bending mode is known to decrease drastically in the solid phase.[13] However, the corresponding spectrum in the O-H stretching range (Figure 2) do not resemble ice-like water, and the frequency of the bending mode is very low, ruling out this explanation. Another explanation for the discrepancy in the bending mode area is a smaller amount of water present in the interlayer space due to the lower hydration enthalpy of $Na^+$. The O-H stretches seen in the spectrum of Na-$Ti_3C_2T_x$ would then have a significant contribution from hydroxyl terminations introduced during etching or dissociated water adsorbed on the surface of the MXene.[14] The complex shape of the O-H stretch region in Na-$Ti_3C_2T_x$ compared to the single crystal $TiO_2$[14] may be explained by the heterogeneity of the surface groups, leading to a large number of different adsorption sites. Sum frequency generation experiments found that the response in the stretching mode region could be simulated both with and without the presence of dissociated interfacial water in addition to liquid water, while, in contrast, the simulated vibrational density of states (VDOS) in the bending mode region decreased drastically when dissociated interfacial water was included.[15] However, X-ray absorption spectroscopic measurements indicate that Na+ intercalated into $Ti_3C_2T_x$ induces a higher oxidation state in Ti compared to $Li^+$, suggesting fewer -OH functional groups in Na-intercalated sample and thus making this explanation less likely.[16] The smaller absorption by the water bending mode vibration for Na-$Ti_3C_2T_x$ could also be due to decreased rotational motion. Calculations for neat $H_2O$ revealed that pure bending motion accounted for only about 30% of the 1650 $cm^{-1}$ VDOS feature, the rest attributed to cross-correlation terms of the bending motion and rotational motion.[17] Confined water in reverse micelles shows slower rotational dynamics,[18] and confined water in $Ti_3C_2T_x$ was determined to be more ordered and less mobile in the order Li < Na < K.[19]

REFERENCES


(1) Smith, D. W. Ionic Hydration Enthalpies. *Journal of Chemical Education* **1977**, 54 (9), 540-542. https://doi.org/10.1021/ed054p540.

(2) Chuntonov, L.; Kumar, R.; Kuroda, D. G. Non-Linear Infrared Spectroscopy of the Water Bending Mode: Direct Experimental Evidence of Hydration Shell Reorganization? *Physical Chemistry Chemical Physics* **2014**, *16* (26), 13172–13181. https://doi.org/10.1039/C4CP00643G.

(3) Brubach, J.-B.; Mermet, A.; Filabozzi, A.; Gerschel, A.; Lairez, D.; Krafft, M. P.; Roy, P. Dependence of Water Dynamics Upon Confinement Size. *The Journal of Physical Chemistry B* **2001**, *105* (2), 430–435. https://doi.org/10.1021/jp002983s.

(4) Ohno, K.; Okimura, M.; Akai, N.; Katsumoto, Y. The Effect of Cooperative Hydrogen Bonding on the OH Stretching-Band Shift for Water Clusters Studied by Matrix-Isolation Infrared Spectroscopy and Density Functional Theory. *Physical Chemistry Chemical Physics* **2005**, *7* (16), 3005–3014. https://doi.org/10.1039/B506641G.

(5) Schmidt, D. A.; Miki, K. Structural Correlations in Liquid Water: A New Interpretation of IR Spectroscopy. *Journal of Physical Chemistry A* **2007**, *111* (40), 10119–10122. https://doi.org/10.1021/jp074737n.

(6) Liu, Y.; Ojamäe, L. Fingerprints in IR OH Vibrational Spectra of $H_2O$ Clusters from Different H-Bond Conformations by Means of Quantum-Chemical Computations. *Journal of Molecular Modeling* **2014**, *20* (6), 2281. https://doi.org/10.1007/s00894-014-2281-x.

(7) Devlin, J. P.; Wooldridge, P. J.; Ritzhaupt, G. Decoupled Isotopomer Vibrational Frequencies in Cubic Ice: A Simple Unified View of the Fermi Diads of Decoupled $H_2O$, HOD, and $D_2O$. *The Journal of Chemical Physics* **1986**, *84* (11), 6095–6100. https://doi.org/10.1063/1.450799.

(8) Devlin, J. P.; Sadlej, J.; Buch, V. Infrared Spectra of Large $H_2O$ Clusters: New Understanding of the Elusive Bending Mode of Ice. *The Journal of Physical Chemistry A* **2001**, *105* (6), 974–983. https://doi.org/10.1021/jp003455j.

(9) Falk, M. The Frequency of the H-O-H Bending Fundamental in Solids and Liquids. *Spectrochimica Acta Part A: Molecular Spectroscopy* **1984**, *40* (1), 43–48. https://doi.org/10.1016/0584-8539(84)80027-6.

(10) Xu, W.; Johnston, C. T.; Parker, P.; Agnew, S. F. Infrared Study of Water Sorption on Na-, Li-, Ca-, and Mg-Exchanged (SWy-1 and SAz-1) Montmorillonite. *Clays and Clay Minerals* **2000**, *48* (1), 120–131. https://doi.org/10.1346/CCMN.2000.0480115.

(11) Kemp, D. D.; Gordon, M. S. An Interpretation of the Enhancement of the Water Dipole Moment Due to the Presence of Other Water Molecules. *The Journal of Physical Chemistry A* **2008**, *112* (22), 4885–4894. https://doi.org/10.1021/jp801921f.

(12) Miller, D. J.; Lisy, J. M. Hydrated Alkali-Metal Cations: Infrared Spectroscopy and Ab Initio Calculations of $M^+(H_2O)_{x=2-5}Ar$ Cluster Ions for M = Li, Na, K, and Cs. *Journal of the American Chemical Society* **2008**, *130* (46), 15381–15392. https://doi.org/10.1021/ja803665q.

(13) Mallamace, F.; Broccio, M.; Corsaro, C.; Faraone, A.; Majolino, D.; Venuti, V.; Liu, L.; Mou, C.-Y.; Chen, S.-H. Evidence of the Existence of the Low-Density Liquid Phase in

Supercooled, Confined Water. *Proceedings of the National Academy of Sciences* **2007**, *104* (2), 424–428. https://doi.org/10.1073/pnas.0607138104.

(14) Takahashi, K.; Yui, H. Analysis of Surface OH Groups on $TiO_2$ Single Crystal with Polarization Modulation Infrared External Reflection Spectroscopy. *The Journal of Physical Chemistry C* **2009**, *113* (47), 20322–20327. https://doi.org/10.1021/jp903426s.

(15) Calegari Andrade, M. F.; Ko, H.-Y.; Car, R.; Selloni, A. Structure, Polarization, and Sum Frequency Generation Spectrum of Interfacial Water on Anatase $TiO_2$. *The Journal of Physical Chemistry Letters* **2018**, *9* (23), 6716–6721. https://doi.org/10.1021/acs.jpclett.8b03103.

(16) Al-Temimy, A.; Prenger, K.; Golnak, R.; Lounasvuori, M.; Naguib, M.; Petit, T. Impact of Cation Intercalation on the Electronic Structure of $Ti_3C_2T_x$ MXenes in Sulfuric Acid. *ACS Applied Materials and Interfaces* **2020**, *12* (13), 15087–15094. https://doi.org/10.1021/acsami.9b22122.

(17) Yu, C.-C.; Chiang, K.-Y.; Okuno, M.; Seki, T.; Ohto, T.; Yu, X.; Korepanov, V.; Hamaguchi, H.; Bonn, M.; Hunger, J.; et al. Vibrational Couplings and Energy Transfer Pathways of Water's Bending Mode. *Nature Communications* **2020**, *11* (1), 5977. https://doi.org/10.1038/s41467-020-19759-w.

(18) Biswas, R.; Furtado, J.; Bagchi, B. Layerwise Decomposition of Water Dynamics in Reverse Micelles: A Simulation Study of Two-Dimensional Infrared Spectrum. *The Journal of Chemical Physics* **2013**, *139* (14), 144906. https://doi.org/10.1063/1.4824446.

(19) Osti, N. C.; Naguib, M.; Ganeshan, K.; Shin, Y. K.; Ostadhossein, A.; Van Duin, A. C. T.; Cheng, Y.; Daemen, L. L.; Gogotsi, Y.; Mamontov, E.; et al. Influence of metal ions intercalation on the vibrational dynamics of water confined between MXene layers. *Physical Review Materials* **2017**, *1* (6), 1–8. https://doi.org/10.1103/PhysRevMaterials.1.065406.
